# Novel circulating microRNAs expression profile in colon cancer: a pilot study

**DOI:** 10.1186/s40001-017-0294-5

**Published:** 2017-11-29

**Authors:** Ya-nan Wang, Zhao-hua Chen, Wei-chang Chen

**Affiliations:** 1 The First Affiliated Hospital of Soochow University, Shizi Street #188, Suzhou, 215006 Jiangsu People’s Republic of China; 2 Suzhou Municipal Hospital, Suzhou Hospital Affiliated to Nanjing Medical University, Suzhou, 215002 Jiangsu People’s Republic of China

**Keywords:** Colon cancer, MicroRNA-gene microarray, Molecular marker expression profile, Genetic diagnosis

## Abstract

**Purpose:**

To identify the expression profile of novel microRNAs (miRNAs) in colon cancer and evaluate their clinical applicability.

**Methods:**

Differences in the expression of serum miRNAs in patients with colon cancer and healthy controls were identified using miRNA microarrays. Differentially expressed miRNAs were verified via real-time polymerase chain reaction (PCR) using sera from 50 patients with colon cancer and 44 healthy controls. These miRNAs were also verified in a double-blind validation experiment using sera from 30 patients with colon cancer, 30 patients with colonic polyps, and 30 healthy controls.

**Results:**

Microarray hybridization revealed that 87 miRNAs were differentially expressed between the sera of patients with colon cancer and healthy controls. Among these miRNAs, 39 miRNAs were up-regulated, whereas 48 miRNAs were down-regulated. Verification of the expression of these miRNAs using real-time PCR revealed that the expression levels of *miR*-*31*, *miR*-*141*, *miR*-*224*-*3p*, *miR*-*576*-*5p*, and *miR*-*4669* were significantly different between patients with colon cancer and healthy controls. Using these five miRNAs to construct a miRNA expression profile (or miRNA panel) will facilitate more effective diagnosis of colon cancer.

**Conclusion:**

Clinical analysis of *miR*-*31*, *miR*-*141*, *miR*-*224*-*3p*, *miR*-*576*-*5p*, and *miR*-*4669* expression in patients with colon cancer may facilitate the diagnosis of colon cancer.

## Background

Colorectal cancer (CRC) is the fifth largest prevalent cancer in China. Since 2002, the incidence of CRC has increased from 16.4 to 34.3 per 10^5^ persons. The development of a positive and effective preventive measure is a major public health concern [1]. Additionally, early diagnosis of cancer in the clinical setting is particularly important [2]. As a new detection technology, genetic diagnosis has very wide application potential in tumor identification, optimization of treatment plan, improving patient prognosis, and tumor monitoring [3]. The expression profile of circulating molecular marker microRNAs (miRNAs) is an important component in tumor identification and genetic diagnosis [4]. miRNAs are non-coding, single-stranded RNAs that are 19–24 nucleotides in length. They are highly conserved, encoded by the host, and widely found in plants and animals. Since the discovery of the first miRNA let4 in *Caenorhabditis elegans* by Lee et al. [5] in 1993, there were 17,000 miRNA sequences and 15,000 miRNA loci belonging to 140 species in the miRbase as of 2011 [6].

Recent evidence has indicated that miRNAs are involved in various cancers [7]. Alterations of miRNA expression has been observed in many human tumors, including colon cancer [8], and these miRNAs are potential molecular markers for the diagnosis of colon cancer. For example, miR-155 was found to promote cancer cell proliferation, migration, invasion, drug resistance, and poor prognosis [9]. The miR-21 can increase proliferation of cancer cells as well as the clinical stage and recurrence rate, and reduce the apoptosis of tumor cells and survival duration of patients [10]. Members of the miR-17-92 family, can increase proliferation of tumor cells by affecting their cell cycle [11]. Moreover, as miR-17-92 is relatively more sensitive, specific, and accurate [based on the area under the receiver operating characteristic (ROC) curve], it could be used as a marker for tumor identification, prognosis evaluation, and recurrence monitoring [12–18]. The potential use of miR-17-92 as a marker sets a new path for the development of microRNA-derived molecular medicine for colon cancer. However, some miRNAs are lowly expressed in colon cancer. For instance, miRNA-145 could inhibit tumor metastasis and invasion, and hence may serve as a marker for early diagnosis of colon cancer [19, 20]. High expression of the Lethal-7 family could inhibit the proliferation of cancer cells and tumor signal pathways to reduce the invasiveness of colon cancer that leads to poor prognosis, and improve the survival duration and progression-free survival time [21]. However, current studies are mostly focused on single miRNA targets for the diagnosis of colon cancer, and the accuracy and efficiency of such studies are low. Following the progress in miRNA research in recent years, the miRNA microarray detection system is thus developed. In this study, miRNA microarrays were used to detect the differential expression of various miRNAs in the sera of colon cancer patients and healthy controls. Real-time PCR was used to evaluate the differentially expressed miRNAs in colon cancer to identify their expression profile in the sera of colon cancer patients. The expression profiles of these miRNAs are of great clinical significance in the identification of colon cancer, the monitoring of tumor development, and evaluation of treatment plans, the prognosis of colon cancer patients, and their recurrence state.

## Methods

### Patients

Three serum samples from hospitalized patients with colon cancer (1 man and 2 women) at the Suzhou Municipal Hospital were used for microarray analysis. At the time of sample collection, none of the patients had undergone surgical treatment, radiotherapy, or chemotherapy. Diagnosis was made by pathological examination, and the histopathological types of the tumors were evaluated based on the pathological stages defined by the World Health Organization (WHO). Three serum samples from healthy controls were collected from patients undergoing physical examinations at the hospital.

For verification of miRNA expression by polymerase chain reaction (PCR), 50 serum samples were collected from patients (25 men and 25 women) with colon cancer at Suzhou Municipal Hospital. At the time of sample collection, the patients had not undergone surgical treatment, radiotherapy, or chemotherapy. Diagnosis was made by pathological examination, and the histopathological types of the tumors were evaluated based on the pathological stages defined by the WHO. Forty-four serum samples from healthy controls were collected from patients undergoing physical examination at the hospital. For the double-blind validation of miRNAs, 30 serum samples from hospitalized patients with colon cancer, 30 serum samples from patients with colon polyps, and 30 serum samples from healthy controls were collected from Suzhou Municipal Hospital. This study was approved by the hospital medical ethics committee, and informed consent was obtained from all patients.

### Sample collection

Serum samples were collected from patients with colon cancer, patients with colon polyps prior to surgery, and healthy volunteers during physical examinations. Fasting venous blood (5 mL) was collected using disposable Vacutainer blood collection tubes. The blood was centrifuged at 3000*g* for 5 min to separate the serum from the blood. The serum (1 mL) was preserved at − 80 °C after centrifugation.

### Microarray analysis

miRNAme microarrays (miRNA UniTag™) were used to test the sera of three patients with colon cancer and three healthy controls. The samples were sent to Suzhou Institute of Nano-tech and Nano-bionics (SINANO) of the Chinese Academy of Sciences (CAS) for detection using the miRNA UniTag microarrays (i.e., unlabeled miRNA microarray analysis) [22]. After microarray hybridization, the data were analyzed. Differentially expressed miRNAs in patients with colon cancer compared with healthy controls were identified as those showing changes in expression of at least 1.5-fold with *P* < 0.05.

### Real-time PCR analysis

For real-time PCR verification of miRNA expression, RNA was extracted from the serum samples. Serum (250 μL) was added to 750 μL TRIzol (Beijing Tiangen Biotech Co., Ltd.) in a 2 mL centrifuge tube, which was then vortexed to ensure complete mixing. RNA extraction was carried out according to the manufacturer’s instructions. The absorbance ratio (A260/280) of total RNA, which was between 1.8 and 2.2, was determined using an ultraviolet (UV) spectrophotometer. The 3′ ends of the miRNAs were modified to have a poly(A) tail, and the miRNAs were reverse transcribed using an miRcute miRNA cDNA First-Strand Synthesis kit (Beijing Tiangen Biotech Co., Ltd.). Real-time fluorescence quantitative PCR was performed using an miRcute miRNA Fluorescence Quantitative Detection kit (Tiangen Biotech Co., Ltd.). Each reaction (20 μL) included 10 μL of 2 × miRcute miRNA Premix (containing SYBR and ROX), 0.4 μL of 10 μM upstream and downstream primers, 2 μL first-strand cDNA, 1.6 μL of 50 × ROX reference dye, and 5.6 μL ddH_2_O. The cycling conditions were as follows: predenaturation at 94 °C for 2 min, followed by 43 cycles of 94 °C for 20 s and 60 °C for 34 s. The expression of *miR*-*16* was used as an internal Ref. [23] for normalization of target gene expression. Triplicate reactions were carried out for each target gene. Expression was calculated using the formula $2^{{ - \Delta \Delta C_{\text{t}} }}$ [24]. Amplified products of the fluorescence quantitative PCR (10 μL) were electrophoresed on 30 g/Lagarose gels, and gels were imaged using a Bio-Rad Gel Doc system (Broad Import & Export Trading Co., Ltd. USA).

### Statistical analysis

SPSS 17.0 (USA) was used for statistical analysis. The one-sample Kolmogorov–Smirnov test was used to evaluate the normal distribution of the data. Data that were normally distributed were evaluated using two-sample *t* tests. One-way analysis of variance (ANOVA) was used to compare multiple groups of data, and SNK-q tests were used to compare two groups. Differences with *P* < 0.05 were considered statistically significant.

## Results

### Patient demographics

The clinicopathological data for the patients are presented in Tables 1 and 2.

**Table 1:** Pathological parameters of three colon cancer serum samples for microarray

Microarray serum sample	Age	Sex	Tumor size (cm)	Differentiation	TNM	Lymphatic metastasis
1#	84	Female	3	Media	III	Positive
2#	64	Male	3	Low	II	Negative
3#	58	Female	5	Media	II	Negative

**Table 2:** Clinical data of patients with colon cancer (for verification)

Parameter	Number
Age	
< 60	18
60–70	19
> 70	13
Sex	
Male	25
Female	25
Tumor size (cm)	
*d* ≤ 4	29
*d* > 4	21
Differentiation	
High	5
Medium	37
Low	8
TNM	
I II	18
III	24
IV	8
Lymphatic metastasis	
Positive	28
Negative	22

### Microarray analysis of miRNA expression profiles in serum from patients with colon cancer

**Fig. 1:**
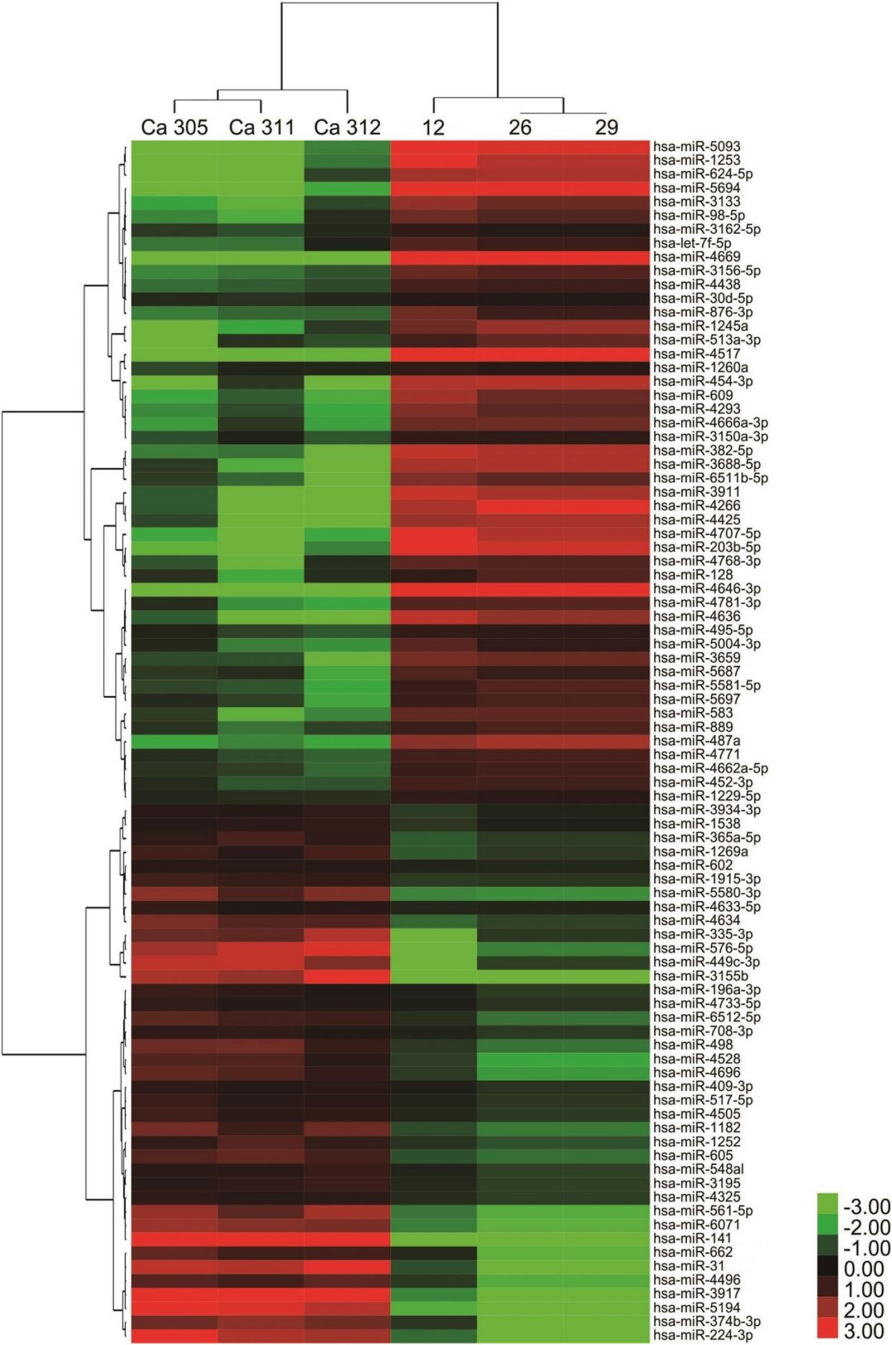
Heat map of the miRNA microarray using serum samples from three patients with colon cancer and three healthy controls. Green denotes miRNAs with low expression, and red denotes miRNAs with high expression. The cut-off parameters were an expression change of at least 1.5-fold and a *P* value of less than 0.05. Ca, CRC serum; T, healthy serum

A total of 87 miRNAs were differentially expressed in the sera of patients with colon cancer compared with that in healthy controls. Among these, 39 miRNAs were up-regulated, and 48 miRNAs were down-regulated. The heat map of the 87 differentially expressed miRNAs is shown in Fig. 1, and the differentially expressed miRNAs are listed in Table 3.

**Table 3:** Differentially expressed miRNAs in patients with colon cancer

Up-regulated miRNAs	Down-regulated miRNAs	
*hsa*-*miR*-*602*	*hsa*-*miR*-*4669*	*hsa*-*miR*-*4771*
*hsa*-*miR*-*4733*-*5p*	*hsa*-*miR*-*4646*-*3p*	*hsa*-*miR*-*4662a*-*5p*
*hsa*-*miR*-*409*-*3p*	*hsa*-*miR*-*4517*	*hsa*-*miR*-*452*-*3p*
*hsa*-*miR*-*3934*-*3p*	*hsa*-*miR*-*5694*	*hsa*-*miR*-*3150a*-*3p*
*hsa*-*miR*-*1538*	*hsa*-*miR*-*5093*	*hsa*-*miR*-*495*-*5p*
*hsa*-*miR*-*4633*-*5p*	*hsa*-*miR*-*1253*	*hsa*-*miR*-*3162*-*5p*
*hsa*-*miR*-*708*-*3p*	*hsa*-*miR*-*3911*	*hsa*-*miR*-*1260a*
*hsa*-*miR*-*4325*	*hsa*-*miR*-*4707*-*5p*	*hsa*-*miR*-*30d*-*5p*
*hsa*-*miR*-*196a*-*3p*	*hsa*-*miR*-*4266*	*hsa*-*miR*-*1229*-*5p*
*hsa*-*miR*-*517*-*5p*	*hsa*-*miR*-*203b*-*5p*	
*hsa*-*miR*-*548al*	*hsa*-*miR*-*4425*	
*hsa*-*miR*-*3195*	*hsa*-*miR*-*624*-*5p*	
*hsa*-*miR*-*4505*	*hsa*-*miR*-*4636*	
*hsa*-*miR*-*365a*-*5p*	*hsa*-*miR*-*454*-*3p*	
*hsa*-*miR*-*1269a*	*hsa*-*miR*-*382*-*5p*	
*hsa*-*miR*-*1915*-*3p*	*hsa*-*miR*-*3688*-*5p*	
*hsa*-*miR*-*1252*	*hsa*-*miR*-*487a*	
*hsa*-*miR*-*4528*	*hsa*-*miR*-*1245a*	
*hsa*-*miR*-*6512*-*5p*	*hsa*-*miR*-*609*	
*hsa*-*miR*-*4696*	*hsa*-*miR*-*3133*	
*hsa*-*miR*-*662*	*hsa*-*miR*-*6511b*-*5p*	
*hsa*-*miR*-*4496*	*hsa*-*miR*-*583*	
*hsa*-*miR*-*605*	*hsa*-*miR*-*4293*	
*hsa*-*miR*-*498*	*hsa*-*miR*-*513a*-*3p*	
*hsa*-*miR*-*1182*	*hsa*-*miR*-*3659*	
*hsa*-*miR*-*4634*	*hsa*-*miR*-*4666a*-*3p*	
*hsa*-*miR*-*5580*-*3p*	*hsa*-*miR*-*3156*-*5p*	
*hsa*-*miR*-*374b*-*3p*	*hsa*-*miR*-*876*-*3p*	
*hsa*-*miR*-*335*-*3p*	*hsa*-*miR*-*98*-*5p*	
*hsa*-*miR*-*6071*	*hsa*-*miR*-*4781*-*3p*	
*hsa*-*miR*-*561*-*5p*	*hsa*-*miR*-*5581*-*5p*	
*hsa*-*miR*-*449c*-*3p*	*hsa*-*miR*-*4768*-*3p*	
*hsa*-*miR*-*224*-*3p*	*hsa*-*miR*-*4438*	
*hsa*-*miR*-*31*	*hsa*-*miR*-*5004*-*3p*	
*hsa*-*miR*-*5194*	*hsa*-*miR*-*5697*	
*hsa*-*miR*-*3917*	*hsa*-*miR*-*5687*	
*hsa*-*miR*-*141*	*hsa*-*let*-*7f*-*5p*	
*hsa*-*miR*-*3155b*	*hsa*-*miR*-*128*	
*hsa*-*miR*-*576*-*5p*	*hsa*-*miR*-*889*	

### Verification of the differential expression of miRNAs using PCR

**Fig. 2:**
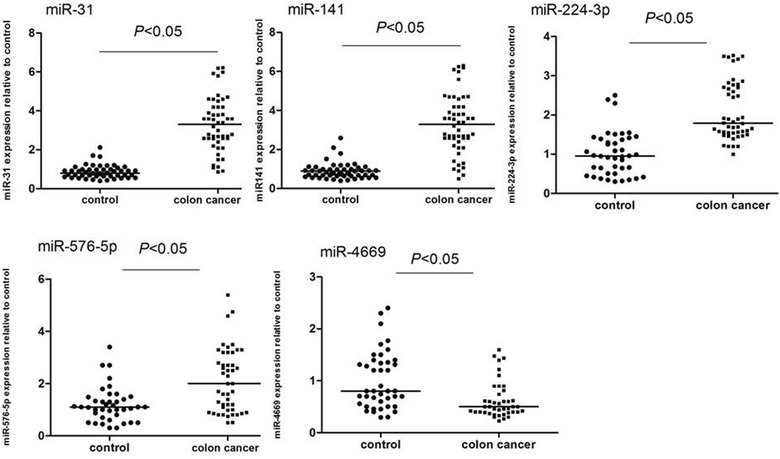
Expression of miRNAs in the colon cancer and control groups

Twelve of the 87 differentially expressed miRNAs identified from microarrays have been shown to be associated with cellular differentiation and metastasis in colon cancer [25]. Thus, the expression of these 12 miRNAs was verified using real-time PCR. From this analysis, we found that the expression levels of *miR*-*31*, *miR*-*141*, *miR*-*224*-*3p*, *miR*-*576*-*5p*, and *miR*-*4669* were significantly different in patients with colon cancer compared with that in healthy controls (Fig. 2).

**Fig. 3:**
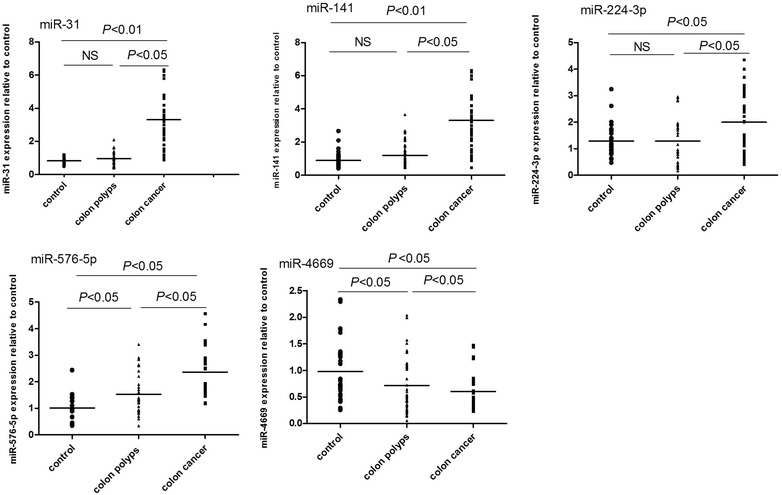
Expression profiles of circulating miRNAs in the serum of patients with colon cancer, patients with colon polyps, and healthy controls

In a double-blind validation, the differential expression of these five miRNAs in colon cancer was consistent with the results of microarray analysis. Thus, these five miRNAs were then used to form an miRNA expression profile (miR panel, as shown in Fig. 3).

### Analysis of the identified miRNAs as markers of colon cancer

**Fig. 4:**
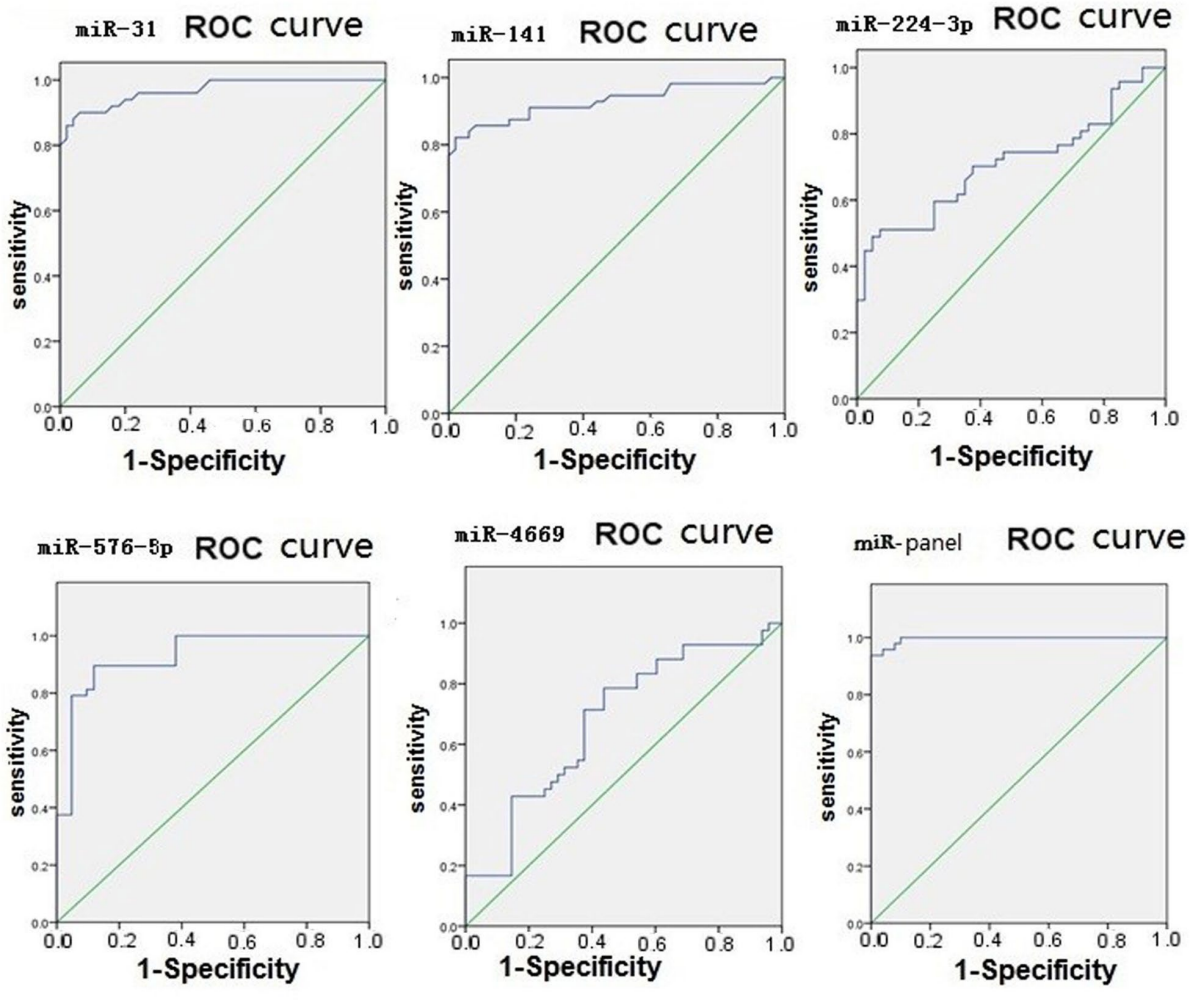
ROC curves for miRNA-based diagnosis of colon cancer (microarrays)

**Fig. 5:**
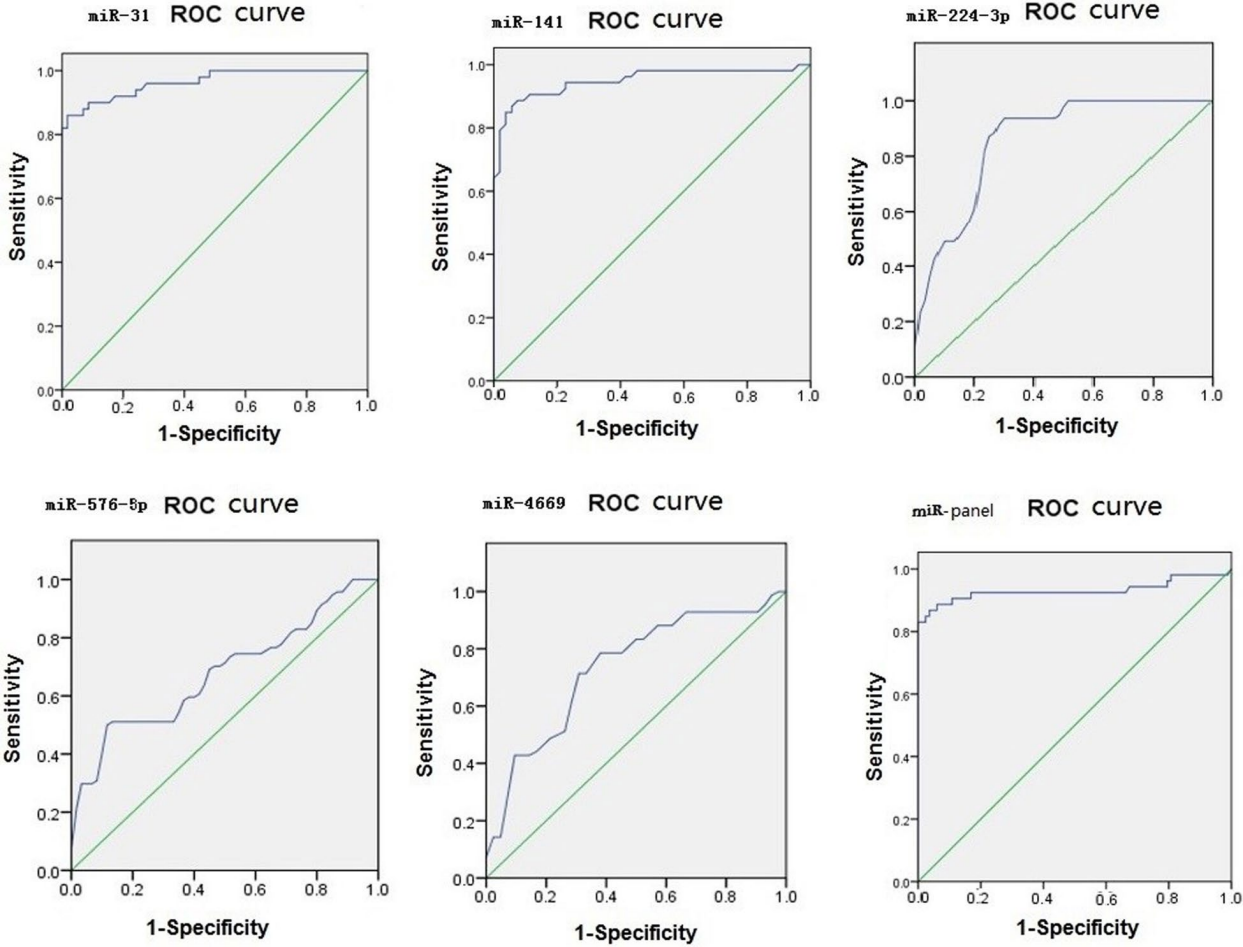
ROC curves for miRNA-based diagnosis of colon cancer (double-blind validation test)

For ROC curve analysis, pathology reports from patients who provided CRC samples were used as an evaluation standard. The ROC curves for miRNA-based diagnosis of colon cancer were plotted (Figs. 4, 5, Tables 4, 5).

**Table 4:** Area under the ROC curve and asymptotic 95% confidence interval for miRNA-based diagnosis of colon cancer (microarrays)

miRNA	Area under the curve	Standard error	Asymptotic significance	Asymptotic 95% confidence interval	
				Upper limit	Lower limit
*miR*-*31*	0.968	0.015	0.000	0.939	0.998
*miR*-*141*	0.929	0.027	0.000	0.876	0.981
*miR*-*224*-*3p*	0.709	0.056	0.001	0.599	0.818
*miR*-*576*-*5p*	0.927	0.026	0.000	0.874	0.982
*miR*-*4669*	0.678	0.057	0.004	0.576	0.790
miR expression profile	0.995	0.004	0.000	0.999	1.000

**Table 5:** Area under the ROC curve and asymptotic 95% confidence intervals for miRNA-based diagnosis of colon cancer in the double-blind validation test

miRNA	Area under the curve	Standard error	Asymptotic significance	Asymptotic 95% confidence interval	
				Upper limit	Lower limit
*miR*-*31*	0.931	0.03	0.001	0.872	0.996
*miR*-*141*	0.949	0.023	0.000	0.904	0.993
*miR*-*224*-*3p*	0.857	0.035	0.000	0.788	0.927
*miR*-*576*-*5p*	0.678	0.054	0.002	0.573	0.783
*miR*-*4669*	0.734	0.055	0.000	0.625	0.842
miR panel	0.964	0.030	0.000	0.872	0.99

From the ROC curves, the miRNA expression profiles (miR panel) obtained from the microarray and double-blind validation tests showed area under the curve values of 0.995 and 0.964, respectively. An miRNA expression profile consisting of the five miRNAs was more effective for predicting the diagnosis of colon cancer than that of a single miRNA.

### Analysis the expression of miRNAs with colon cancer different characteristics

**Fig. 6:**
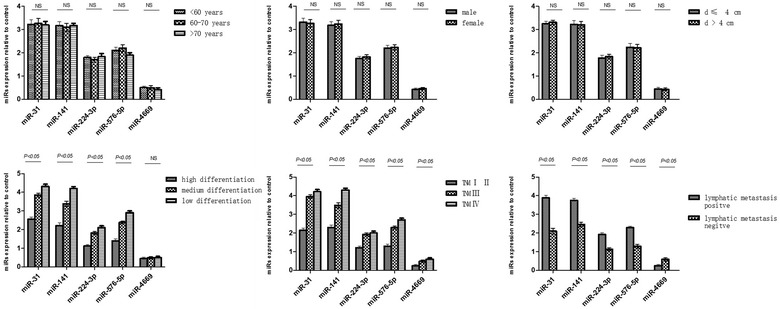
Expression of miRNAs in colon cancer different characteristics

We also investigated the expression of miRNAs upon the various pathological parameters in colon cancer (see Fig. 6). There was significant difference of miR-31, miR-141, miR-224-3p, and miR-576-5p between well-differentiated, moderate-differentiated, and poor-differentiated colon cancer (*P* < 0.05). It was obvious that the expression of above-mentioned RNAs was of increase with the differentiation decreased. In addition, miR-31, miR-141, miR-224-3p, miR-576-5p, and miR-4669 showed notably different expression during different TNM stages (*P* < 0.05). Note that, miR-31, miR-141, miR-224-3p, and miR-576-5p displayed high expression in lymphatic metastasis colon cancer, but miR-4669 exhibited enhancement on expression only in non-lymphatic metastasis colon cancer.

## Discussion

CRC is one of the most common types of malignant tumors. The development of CRC involves the stages of hyperplasia, adenoma, and carcinogenesis, and this pathogenic process involves multiple genes. In recent years, the role of miRNAs in the development of CRC has become a major research focus. Current studies have indicated that miRNAs could function as oncogenes and tumor suppressors in CRC. Moreover, the development of miRNA microarrays has improved studies of the pathogenesis, early diagnosis, and prognosis of tumors owing to the advantages of high-throughput analysis and rapid detection of miRNA expression.

The UniTag microarray used in this study is an unlabeled miRNA microarray detection platform (including 2154 miRNAs, version 19.0) developed by the SINANO of CAS. The base stacking hybridization technology and the universal tag used by this platform can distinguish different miRNAs having single nucleotide differences. Moreover, this approach is particularly effective for ruling out inactive pri-miRNAs and pre-miRNAs that could yield cross-hybridized signals [22]. Our microarray screen revealed that 87 miRNAs were differentially expressed in the serum of patients with colon cancer compared with that of healthy controls. Among these miRNAs, 39 miRNAs were up-regulated, and 48 miRNAs were down-regulated, with significant differences in the expression of greater than 1.5-fold. Furthermore, real-time PCR verified the changes in the expression of 5 of 12 miRNAs known to be related to cellular differentiation and metastasis of colon cancer [25]; specifically, *miR*-*31*, *miR*-*141*, *miR*-*224*-*3p*, *miR*-*576*-*5p*, and *miR*-*4669* were found to be differentially expressed in patients with colon cancer compared with that in healthy controls. Notably, *miR*-*31* and *miR*-*141* expression levels were three-fold higher in patients with colon cancer than in healthy controls. Recent studies have reported the close correlation between *miR*-*31* and CRC. For example, Yang et al. found that *miR*-*31* was highly expressed in CRC tissues and that *miR*-*31* expression was significantly higher in metastasized CRC tissues than in non-metastasized CRC tissues [26]. However, the specific role of *miR*-*31* in CRC is unknown. Increased expression of *miR*-*31* in serum suggested that *miR*-*31* could serve as a potential tumor marker, and anticancer treatment targeting *miR*-*31* could represent another important treatment option for CRC. *miR*-*141* is evolutionarily conserved and shows high expression in epithelial tissues. *miR*-*141* has been shown to be up-regulated in ovarian cancer, lung cancer, cholangiocarcinoma, prostate cancer, and CRC but down-regulated in gastric cancer, pancreatic cancer, liver cancer, and breast cancer, consistent with the ability of miRNAs to have oncogenic or tumor-suppressive roles indifferent of tumor types [27]. Additionally, *miR*-*141* expression has been shown to be higher in the blood of patients with late-stage CRC than that in the blood of patients with middle-stage CRC [28]. The results of this study indicated that *miR*-*141* was highly expressed in the serum of patients with colon cancer and had an area under the ROC curve of 0.93 for the diagnosis of colon cancer, indicating relatively high diagnostic efficacy. Moreover, the expression levels of *miR*-*576*-*5p* and *miR*-*4669* were significantly associated with the differential diagnosis of colonic polyps and colon cancer.

Importantly, the differential expression of these five miRNAs in patients with colon cancer was confirmed in a double-blind validation experiment, and these five miRNAs were then used to form an miRNA expression profile (miR panel). By plotting ROC curves, we found that the area under the curve values for the miR panel in the initial microarray screen and the double-blind validation experiment were 0.995 and 0.964, respectively, demonstrating that the miR panel showed higher diagnostic accuracy for colon cancer than that of single miRNA expression. Because serum collection is convenient and simple, analysis of serum miRNA levels may be a suitable clinical tool for diagnosing and evaluating the prognosis of colon cancer. Indeed, the use of serum miRNA analysis may improve patient comfort during colon cancer evaluation and increase the sensitivity and specificity for the early diagnosis of colon cancer. According to the comparison of miRNAs among different pathologic parameters in colon cancer, we observed that the expression of miR-31, miR-141, miR-224-3p, miR-576-5p was inversely correlated with the differentiation of cancer. miR-31, miR-141, miR-224-3p, miR-576-5p showed higher expression at TNM III and TNM IV than TNMI/II, miR-4669 was opposite. Furthermore, miR-31, miR-141, miR-224-3p, and miR-576-5p were commonly observed in lymphatic metastasis colon cancer, contrariwise, miR-4669 expressed highly only in non-lymphatic metastasis colon cancer. Therefore, the analysis of serum miRNA levels also can enhance prognosis evaluation, and prolong the survival of patients with colon cancer. Thus, after further studies to overcome the issues associated with high costs, miRNAme microarrays may be powerful tools for tumor diagnosis and treatment.

## Conclusion

Our results suggested that analysis of the expression profiles of *miR*-*31*, *miR*-*141*, *miR*-*224*-*3p*, *miR*-*576*-*5p*, and *miR*-*4669* may facilitate the diagnosis of colon cancer. The expression profiles of these miRNAs may be potential important markers for clinical evaluation of colon cancer.

## Abbreviations

CRC: colorectal cancer
PCR: polymerase chain reaction
miRNAs: microRNAs
ROC: receiver operating characteristic
